# Anti-Neutrophil Cytoplasmic Antibody in Behçet’s Disease

**Published:** 2006-02

**Authors:** Nursen Duzgun, Mehmet Sahin, Ergin Ayaslioglu

**Affiliations:** 1 *Department of Clinical Immunology and Rheumatology, Ankara University School of Medicine Ankara, Turkey;*; 2 *Department of Internal Medicine, Süleyman Demirel University School of Medicine, Isparta, Turkey;*; 3 *Department of Infectious Diseases and Clinical Microbiology, Kirikkale University School of Medicine, Kirikkale, Turkey*

**Keywords:** ANCA, behçet’s disease, vasculitis

## Abstract

Anti neutrophil cytoplasmic antibody (ANCA) is strongly associated with some vasculitic disorders. Behçet’s disease (BD) is a systemic vasculitis of unknown etiology. In this study, ANCA was found to be positive in 8 out of 66 patients (10.2%) with BD by combination testing consisting of immunofluorescence and ELISA [one patient showed an atypical pattern by indirect immunofloresence techique, 6 patients were reactive to bacterial-permeability increasing protein (BPI) and one patient was reactive to Cathepsin G in ELISA]. There were no vascular manifestations such as veneous or arterial thrombosis and arterial aneurysms in ANCA-positive patients with BD. The results suggest that ANCA may be found in a minority of BD as those in previous published studies.

## INTRODUCTION

Anti-neutrophil cytoplasmic antibodies (ANCA) are strongly associated with some vasculitic disorders. In systemic vasculitis, interactions between neutrophils and ANCA may initiate endothelial and vascular injury. The presence of ANCA has been established as serological marker especially in the ANCA-associated vasculitis such as Wegener granulomatosis, Churg-Strauss syndrome, microscopic polyangiitis and renal–limited glomerulonephritis (1).

Behçet’s disease (BD) is a chronic, relapsing and multisystem inflammatory disorder with mucocuteneous, ocular, vascular, articular, gastrointestinal and central nerveous system manifestations of unknown etiology (2, 3). The main histological finding of BD is vasculitis both in small and large blood vessels. Histopathology of superficial thrombophlebitis and deep vein thrombosis revealed vasculitis infiltrated lymphocytes and neutrophils (4). The studies have also shown the presence of leucocytoclasis and necrotizing vasculitis (5), increased chemotaxis and phagocytosis (6) and crescentic glomerulonephritis (7) in BD. Neutrophils play an important role in inflammatory response by overproduction of monocyte derived TNF-α, IL-6 and IL-8 (8). Several studies investigated ANCA to assess the role of polymorphonuclear leucocytes in BD (9-11).

By indirect immunofluorescence techique (IIFT) on ethanol-fixed neutrophils, at least two major patterns of ANCA can be identified: first, the cytoplasmic pattern (cANCA) which reflects the presence of anti-proteinase 3 (PR3) antibodies and second, the perinuclear pattern (pANCA) which is mainly produced by anti-myeloperoxidase (MPO) antibodies. PR3 and MPO are contained in cytoplasmic granules and induce the development of ANCA. ANCA against other antigens such as lactoferrin (LF), cathepsin G (Cat.G), elastase (EL), lysozyme (LY) and bactericidal/permeability-increasing protein (BPI) can be identified by ELISA (12). ANCA are mostly tested for their anti-PR3 and anti-MPO activities by either IIFT alone or both by IIFT and ELISA.

We aimed at detecting the presence of ANCA and their clinical importance in the patients with BD, via making an assessment of their reactivity to PR3, MPO and other antigens including LF, Cat.G, EL, LY and BPI using both IIFT (pANCA, cANCA) and ELISA.

## MATERIAL AND METHODS

### Study population

This study included 66 patients (29 females with the mean age of 33.51+/- standard deviation (SD) 9.86 and 37 males with the mean age of 33.74+/- SD 9.91, range 15-56 years) and 20 healthy controls (13 females with the mean age of 28.51+/- 9.57 (SD) and 7 males with a mean age of 29.14+/-3.18 (SD), range 18- 53). All patients fulfilled the International Study Group criteria for the diagnosis of BD (3). Disease duration was 5.7+/-4.8 years. Clinical symptoms of the patients are seen on Table 1. Thirteen patients were on prednisone, 5 patients were on combination of these and other patients were on colchicine. Nine patients without symptom had no therapy.

**Table 1:** Clinical symptoms* of patients with BD (n=66)

Symptom	n (%)

Oral ulcers	57 (86.36)
Genital ulcers	17 (25.75)
Uveitis	12 (18.18)
Eritema nodosum-like lesions	11 (16.66)
Trombophlebitis	7 (10.60)
Neurologic involvement	1 (1.51)
No symptom	9 (13.63)
Positive Pathergy test (at the time of diagnosis)	28 (42)

* Data at the time of blood collection

### ANCA testing: IIFT

Antibodies against granulocytes (c-ANCA / p-ANCA) were analysed by IIFT using a BIOCHIP combination of ethanol-fixed as well as formalin-fixed granulocytes and primate liver in sixty-six sera samples from patients with BD and twenty sera of healthy group. Biochip technology: thin glass slides coated with biological substrates are cut into millimetre-sized fragments (biochips) on a machine. Biochips containing ethanol-fixed human granulocytes are used as a standart substrate for immunofluorescence. Since antibodies against cell nuclei can hide a p-ANCA pattern, an other biochip containing form-aldehyde fixed granulocytes is added, allowing the detection of a part of the p-ANCA, particularly antibodies against MPO, whereas nearly all antinuclear antibodies are completely suppressed. The differentiation between p-ANCA and antibodies against cell nuclei in IIFT can be difficult in some cases, therefore a biochip coated with primate liver is used as additional substrate. It is possible to identify the nuclei of the hepatocytes and the granulocytes in the sinusoids: the nuclei of the neutrophils fluoresce with much more intensity than the hepatocyte nuclei. The following titers were regarded as positive: c-ANCA>1:32, p-ANCA>1:10.

### ANCA testing: ELISA

These sera were tested for autoantibodies against PR3, MPO, LF, Cat.G, EL, LY and BPI using ELISA. The ELISA test kit contains microtiter strips each with 8 reagent wells separately coated with these antigens. The first reaction step:diluted patient samples were incubated with the wells. If the sample is positive, specific antibodies bind to the antigens. A second step: the bound antibodies were detected using peroxidase-labelled anti-human antibodies, which gives a color reaction. The intensity of the formed colour is proportional to the concentration of the corresponding antibodies. Results are shown as “negative” or in “positive” cases, in RU/ml. The cut-off is 20 RU/ml.

### Results

p-ANCA by IIFT was found to be negative in all sera whereas c-ANCA atypical pattern was detected in one patient (1/66 in IFTT). The 6 of 66 sera had antibodies against BPI, only one serum was positive for antibody against cathepsin G by ELISA (7/66 in ELISA). Therefore ANCA positivity was 8/66 (10.2%) in this study (Table 2) Antibodies against other granulocyte antigens were not found by ELISA. Healthy control group was ANCA-negative both by ELISA and IIFT.

**Table 2:** Positive ANCA specifities and values in patients with BD (8/66)

Method	Specifity/Ig	Values (RU/ml)

ELISA	BPI/IgG	80
		187
	BPI/IgA	232
		233
		173
		46
	Cat.G	100
IIFT	Atypical Cytoplasmic pattern (n=1)	
	Total 8 cases (102%)	

## DISCUSSIONS

The development of ANCA-positive small vessel vasculitis depends on an auto- immune response. Once auto-antibodies develop, they may activate neutrophils and monocytes and injure endothelial cells. Other stimuli such as infection or environmental exposure are known to contribute to the induction of ANCA mediated vessel inflammation (13).

The etiology of BD is still unknown and both viral and autoimmune mechanisms have been proposed. Some immune abnormalities such as the functional aberration of T-cell subsets and mild overactivity of B-cells (14, 15) with the presence of autoantibodies such as anti-endothelial cell antibodies and anti-cardiolipin antibodies have been reported in BD (16, 17). These antibodies may contribute to the development of vascular damage and neutrophils are implicated in the pathogenesis of BD (18).

ANCA have rarely been found in patients with BD. In a cohort of 28 patients with BD, only one patient had ANCA-positive by IIFT (10). Our observation that only one serum showed atypical ANCA-positivity by IIFT, is in line with previous studies. Ben Hmida *et al*. did not find detectable ANCA in 46 active and inactive patients with BD (11). ANCA associated vasculitis and renal failure was reported in a female (19) and a male patient (20) with BD. In another study, ANCA were evaluated by IIFT and ELISA for anti-PR3 and anti-MPO antibodies and ANCA were found in three out of 29 patients (10%), mainly those with vasculitis (21). The results of the present study also show that the sera from 8 out of 66 patients (10.2 %) had ANCA positivity (1 in IIFT, 7 in ELISA) and that there were no vascular manifestations in these patients. Six out of 7 patients were reactive to BPI in ELISA. The patients showing BPI-ANCA positivity may have negative IIFT-ANCA results (22-24) as those in our findings. Burrows *et al*. (10) have previously described a patient with BD who exhibited the presence of antibodies to BPI as also unusual cutaneous manifestations. This article reports for the first time, some association of BD with anti-BPI ANCA. However, the presence of BPI-ANCA has previously been reported in the case of chronic infections such as chronic bronchitis or cystic fibrosis (22, 23) and during chronic inflammation such as inflammatory bowel disease (24).

Our data suggest that ANCA positivity may be found probably as a non-spesific finding in a minority of the patients with BD.

## References

[1] Gross WL, Csernok E, Helmchen U (1975). Antineutrophil cytoplasmic antibodies, autoantigens, and systemic vasculitis. APMIS.

[2] Behçet H (1937). Uber rezidivirende, aphthose, durch ein Virus verursachte Geschwure am Mundam Auge und den cenitalien. Dermatol Wochenschr.

[3] International Study Group for Behçet’s Disease (1992). Evalution of diagnostic classification ‘criteria in Behçet’s disease – towards internationally agreed criteria. Br. J. Rheumatol.

[4] Matsumoto T, Uekusa T, Fukuda Y (1991). Vascular- Behçet’s disease A pathologic study of eight cases. Hum. Pathol.

[5] Nazarro P, Mmoncelli P, Nazarro (1966). Cutaneous manifestations of Behçet’s disease. Clinical and histopathological findings. Behçet’s Disease.

[6] Sobel JD, Haim S, Obedean VM, Vesnulam P (1977). Polymorponuclear leucocyte function in Behçet’s disease. J. Clin. Pathol.

[7] Donnely S, Jothy S, Barre P (1989). Crescentic glomerulonephritis in Behçet’s syndrome. Clin. Nephrol.

[8] Mege JL, Dilsen N, Sanguedolce V, Gul A (1993). Overproduction of monocyte derived tumor necrosis factor α, interleukin (IL) 6, IL-8 and increased neutrophil superoxide generation in Behçet’s disease. A comparative study with familial Mediterranean fever and healthy subjects. J. Rheumatol.

[9] Hamza M, Meyer O (1990). The negative antineutrophil cytoplasmic antibodies in Behçet’s disease. Letter Ann. Rheum.

[10] Burrows NP, Zhao MH, Norris PG, Lockwood CM (1996). ANCA associated with Behçet’s disease. J. R. Soc. Med. (Suppl).

[11] BenHmida M, Hachicha J, Kaddour N, Makni H (1997). ANCA in Behçet’s disease. Nephrol. Dial. Transplant.

[12] Wiik A Rasmussen, Wieslander J, van Venrooji WJ, Maini RN (1993). Methods to detect autoantibodies to neutrophilic granulocytes. Manual of biological markers of disease.

[13] Falk RJ, Nachman PH, Jenette JC (2000). Immunopathogenesis of small vessel vasculitis. Clin. Exp. Immunology.

[14] Suzuki N, Sakane T, Uedo Y, Tsunematsu T (1986). Abnormal B cell function in patients with Behçet’s disease. Arthritis Rheum.

[15] Lehner T, Lavery E, Smith R, van der Zee R (1991). Association between the 65-kilo dalton heat shock protein, Streptococcus sangius and the corresponding antibodies in Behçet’s syndrome. Infect Immunol.

[16] Aydintuğ AO, Tokgöz G, D’Cruz D, Gürler A (1993). Antiendothelial cell antibodies in patients with Behçet’s Disease. Clinic. Immunol. Immunopathol.

[17] Tokay S, Direskeneli H, Yurdakul S, Akoğlu T (1996). IgA anticardiolipin antibodies in Behçet’s disease. Rveu. du Rheumatisme. (July-September).

[18] Inoue C, Itoh R, Kawa Y, Mizoguchi M (1994). Pathogenesis of mucocutaneous lesions in Behçet’s disease. J. Dermatol.

[19] Yang CW, Park IS, Kim SY, Chang YS (1993). Antineutrophil cytoplasmic antibody associated vasculitis and renal failure in Behçet’s disease. Nephrol. Dial. Transplant.

[20] Khan IH, Catto GRD, Macleod AM (1994). Antineutrophil cytoplasmic antibody assocaited vasculitis and renal failure in Behçet’s disease. Nephrol. Dial. Transplant.

[21] Vaipoulos G, Hatzinicolaou P, Tsiroyanni A, Mavropulos S (1994). Antineutrophil cytoplasmic in Adamantiades-Behçet’s disease. Br. J. Rheumatol.

[22] Kobayashi O (1998). Clinical role of autoantibody against bactericidal/permeability-increasing protein in chronic airway infection. J. Infect Chemother.

[23] Zhao MH, Jayne DRW, Ardiles LG, Gulley F (1996). Autoantibodies against bactericidal/ permeability-increasing protein in patients with cystic fibrosis. Q. J. Med.

[24] Stoffel MP, Csernok E, Herzberg C, Johnson T (1996). Anti-neutrophil cytoplasmic antibodies (ANCA) directed against bactericidal/permebility increasing protein (BPI): A new seromarker for inflammatory bowel disease and associated disorders. Clin. Exp. Immunol.

